# *In Situ* Investigation of Under-Deposit Microbial Corrosion and its Inhibition Using a Multi-Electrode Array System

**DOI:** 10.3389/fbioe.2021.803610

**Published:** 2022-01-10

**Authors:** Erika M. Suarez, Kateřina Lepková, Maria Forsyth, Mike Y. Tan, Brian Kinsella, Laura L. Machuca

**Affiliations:** ^1^ Curtin Corrosion Centre (CCC), Western Australia School of Mines: Minerals, Energy and Chemical Engineering, Curtin University, Perth, WA, Australia; ^2^ Institute for Frontier Materials and School of Engineering, Deakin University, Geelong, VIC, Australia

**Keywords:** local electrochemistry, under-deposit corrosion, microbiologically influenced corrosion, corrosion inhibition, multi-electrode arrays, under-deposit microbial corrosion

## Abstract

Carbon steel pipelines used in the oil and gas industry can be susceptible to the combined presence of deposits and microorganisms, which can result in a complex phenomenon, recently termed under-deposit microbial corrosion (UDMC). UDMC and its inhibition in CO_2_ ambiance were investigated in real-time using a multi-electrode array (MEA) system and surface profilometry analysis. Maps from corrosion rates, galvanic currents, and corrosion potentials recorded at each microelectrode allowed the visualization of local corrosion events on the steel surface. A marine bacterium *Enterobacter roggenkampii*, an iron-oxidizing, nitrate-reducing microorganism, generated iron deposits on the surface that resulted in pitting corrosion under anaerobic conditions. Areas under deposits displayed anodic behavior, more negative potentials, higher corrosion rates, and pitting compared to areas outside deposits. In the presence of the organic film-forming corrosion inhibitor, 2-Mercaptopyrimidine, the marine bacterium induced local breakdown of the protective inhibitor film and subsequent pitting corrosion of carbon steel. The ability of the MEA system to locally measure self-corrosion processes, galvanic effects and, corrosion potentials across the surface demonstrated its suitability to detect, evaluate and monitor the UDMC process as well as the efficiency of corrosion inhibitors to prevent this corrosion phenomenon. This research highlights the importance of incorporating the microbial component to corrosion inhibitors evaluation to ensure chemical effectiveness in the likely scenario of deposit formation and microbial contamination in oil and gas production equipment.

## 1 Introduction

Microorganisms have contributed to our planet’s evolution over the past four billion years and made it a livable place for larger forms of life (Konhauser, 2006). Iron-oxidizing bacteria (FeOB) have played an essential role in the geochemical evolution of the Earth and nowadays continue influencing terrestrial and aquatic environments. Indeed, in a recent study, it was suggested that iron-oxidizing, nitrate-reducing bacteria could exist in early Martian environments (Price et al., 2018). Ehrenberg who discovered an iron bacteria which he named *Gaillonella ferruginea* (reviewed byPringsheim (1949)] introduced the concept of microorganisms involved in the geological process of iron oxidation early in the 19th century. Later, in the second half of the 19th century, Winogradsky, a founder of modern microbiology, determined that some bacteria could oxidize iron at near-neutral pH [reviewed by Dworkin and Gutnick (2012)]. Since then, this fundamental biological process has inspired microbiologists and geoscientists to focus on the role of metal-oxidizing microorganisms in the biogeochemistry of iron and other elements like manganese.

Microorganisms with these ancient metabolic capabilities are also ubiquitous in seawater and oilfield systems, and their activities have been associated with microbiologically influenced corrosion (MIC) (Magot, 2005; Ollivier and Cayol, 2005). Therefore, biological iron oxidation has also gained attention within the corrosion community. Particularly, because of the increasing demand for nitrate injection as a mitigation strategy against souring of reservoirs in oil and gas fields. The nitrate benefits the proliferation of nitrate-reducing bacteria (NRB), which consume organics available in the reservoir and, therefore suppress the sulfate-reducing bacteria (SRB) population; this competition leads to a decrease of biogenic H_2_S produced by SRB (Coombe et al., 2004; Hubert and Voordouw, 2007; Bodtker et al., 2008; Gieg et al., 2011). However, there are growing concerns about a possible undesired effect; if the injected nitrate is not entirely consumed and then transported through the pipelines, it could lead to MIC by nitrate-reducing microorganisms. There are also concerns in sour wells that use nitrate injection where the production of nitrite (NO_2_^−^) can lead to the formation of elemental sulfur if any H_2_S is present. Elemental sulfur represents a serious issue because it is extremely corrosive to carbon steel.

Some members of the Proteobacteria phylum can oxidize iron and couple it to nitrate reduction in anaerobic environments, instead of oxygen reduction. The iron-oxidizing, nitrate-reducing bacteria, abbreviated as FeONRB, use ferrous iron (Fe^2+^) as electron donor and nitrate (NO_3_^−^) as electron acceptor with organic cosubstrates (Straub et al., 2001; Straub et al., 2004; Straub, 2011). The possibility of the latter being an energy-yielding reaction depends on the redox potential of the Fe^2+^/Fe^3+^ couple being more negative (+200 mV) than the nitrate/nitrite couple (+430 mV), which restricts its metabolism to environments that have circum-neutral or higher pH values (Hedrich et al., 2011). The final metabolic by-products of this anaerobic respiration can be either nitrite (NO_2_^−^), nitric oxide (NO), nitrous oxide (N_2_O) or, dinitrogen gas (N_2_) in a process called denitrification. Also, some FeONRB members can reduce nitrate (NO_3_^−^) to ammonium (NH_4_^+^) (Madigan et al., 2014).

The oxidation of ferrous iron (Fe^2+^) to ferric iron (Fe^3+^) can lead to Fe^3+^ precipitation and accumulation in the form of ochre-like deposits (Hedrich et al., 2011). The formation of Fe^3+^ mineral deposits by bacteria was described by Kappler and Straub (2005) as follows: 1) the initial abiotic oxidation of Fe^2+^ to form mono- and dinuclear dissolved species of Fe^2+^ [FeOH]^2+^ and [Fe_2_(OH_2_)]^4+^; 2) these dissolved species are transformed into polymeric Fe^3+^ colloids; 3) these colloids precipitate to poorly crystalline ferrihydrite; 4) ferrihydrite conversion to either hematite or goethite depending on the reaction conditions. Finally, there is fast precipitation of the Fe^3+^ by-products near the microbial cells due to their low solubility at neutral pH (Miot et al., 2009).

To date, very limited work has been conducted to link the FeONRB biological process to corrosion under anaerobic conditions and much less research related to the effect of biogenic deposits on under-deposit corrosion (UDC). UDC has been recognized as a serious problem in oil and gas production and transportation facilities, accounting for a significant fraction of localized corrosion at otherwise non-corrosive conditions. Stationary deposits can also provide shelter to bacteria, creating conditions that are conducive for MIC. The combined presence of deposits and microorganisms is known to result in a rather complex phenomenon recently termed under-deposit microbial corrosion (UDMC) (Suarez E. et al., 2019). UDMC has been previously reported in both MIC experiments (Mosher et al., 2014; Machuca et al., 2017; Wang and Melchers, 2017; Suarez et al., 2019b) and case studies of pipeline failures (Skovhus et al., 2014; Esan et al., 2001). To date, the causative mechanism of UDMC on carbon steel is still not fully understood.

In general, corrosion mitigation of carbon steel involves chemical treatment with corrosion inhibitors (CIs) and biocide chemicals. However, CI performance is known to be affected by the presence of deposits, e.g., by adsorption of inhibitor compounds on deposits resulting in inhibitor depletion and therefore, insufficient level of protection to the underlying metal surface (Binks et al., 2011; Pandarinathan et al., 2011; Pandarinathan et al., 2013; Suarez et al., 2019c). Additionally, biocide efficiency can be compromised in the presence of deposits, which can provide a shield to bacteria from antimicrobial compounds, facilitating microbial proliferation. Thus, understanding the performance of CIs in the presence of deposits is critical and remains an industry challenge. In particular, CI efficiency under UDMC conditions has rarely been studied. Regular cleaning with pigs is an effective means of controlling MIC and UDC but in some cases, the pipelines cannot be pigged.

Multi-electrode arrays (MEA), can provide temporal and spatial information about galvanic effects taking place on a metal surface in a corroding environment. For instance, visualizing local electrochemical events on the metal surface in non-homogeneous surfaces, such as those created by biofilms and deposits (Tan et al., 2011; Tan and Revie, 2012; Zhang et al., 2014). MEA systems have been used to study general and localized corrosion (Sun and Yang, 2009; Tan, 1998; Tan, 2009; 2011), erosion-corrosion (Xu and Tan, 2018; 2019), coating evaluation (Varela et al., 2015; Mahdavi et al., 2016; Tan et al., 2017), UDC, and corrosion inhibition (Muster et al., 2009; Turnbull et al., 2009; Hinds and Turnbull, 2010; Tan et al., 2011; Zhang et al., 2014) and most recently, to study MIC (Dong et al., 2011; Liu et al., 2019).

This study aims to investigate UDMC by *Enterobacter roggenkampii*, a FeONRB isolated from an oil production facility in Western Australia (Salgar-Chaparro et al., 2020). The inhibition of UDMC by *E. roggenkampii* was evaluated using an organic, film-forming CI, 2-mercaptopyrimidine (MPY). Local galvanic currents, corrosion potentials, and corrosion rates from the MEA system were mapped, and profilometry analysis was used to assess localized corrosion in the presence of the bacterium and the CI. Intracellular adenosine triphosphate (cATP) measurements and bacterial cell counts were also carried out to monitor bacterial growth and activity throughout the exposure. Visualizing *in situ* the UDMC process in the presence and absence of a corrosion inhibitor compound will aid in understanding the complex mechanism of UDMC and the efficiency of corrosion inhibitors under UDMC conditions. This method has shown great promise as a reliable tool for the evaluation and selection of CIs formulated to prevent localized corrosion in industrial facilities.

## 2 Materials and Methods

### 2.1 Test Solution

The test solution for both bacterial growth (seed cultures) and corrosion studies consisted of artificial seawater (ASW) 2.45% salinity containing ammonium nitrate as the only soluble electron acceptor for the bacterium and acetate as a cosubstrate. The ASW composition was as follows: sea salts (Sigma Aldrich) 20 g/L, CH₃COONa 20 mM, NH₄NO_3_ 14 mM and, 0.939 L of ultrapure water (Milli-Q system, resistivity 18.5 MΩ cm). The compounds were mixed and sterilized by autoclaving. The ASW was sparged with a gas mixture of 20% CO_2_ in N_2_ gas (1 bar) for 2 h followed by the addition of 10 ml of vitamins solution and, 1 ml of Wolfe’s mineral elixir (Beech et al., 1999). Finally, the pH was adjusted to 7.0 ± 0.2 using a sterile and de-oxygenated NaHCO_3_ solution (47.62 mM). For the test with CI, MPY (Sigma-Aldrich) was evaluated at a concentration of 0.9 mM. The MPY concentration was selected based on previous work where this CI performed efficiently at deposited-carbon steel surfaces (Pandarinathan et al., 2013). To our knowledge, this CI has not been evaluated in the presence of microorganisms. MPY was added to a separate test solution to allow the compound to dissolve before pumping into the MEA reactor.

### 2.2 Bacterium Preparation

*E. roggenkampii* was maintained at 40°C in anaerobic glass vials. A preliminary test was performed to determine the iron oxidation and nitrate reduction capability of this isolate before the UDMC experiments. For this, the bacterium was grown in 100 ml glass vials containing ASW sparged with CO_2_/N_2_ gas mixture (Section 2.1) and 0.75 cm^2^ carbon steel coupons. The ASW was not supplemented with an external soluble iron source. The bacterial activity was evidenced by iron precipitation and deposition on steel coupons compared to the controls (no bacteria) where no deposits were formed.

For immersion testing using the reactor, the bacterium was inoculated at 10% into an anaerobic test solution and incubated at 40°C for 96 h (exponential phase, 10^8^ cell/mL). Bacterial cells were harvested by centrifugation (3,600 × g, 30 min at room temperature) to remove iron precipitates formed by bacterial activity in the culture. The supernatant was discarded and, the pellet was suspended in 1 ml of sterile oxygen-free ASW. The bacterial cell suspension was then inoculated into the reactor containing the anaerobic test solution.

### 2.3 Tests Materials

**FIGURE 1:**
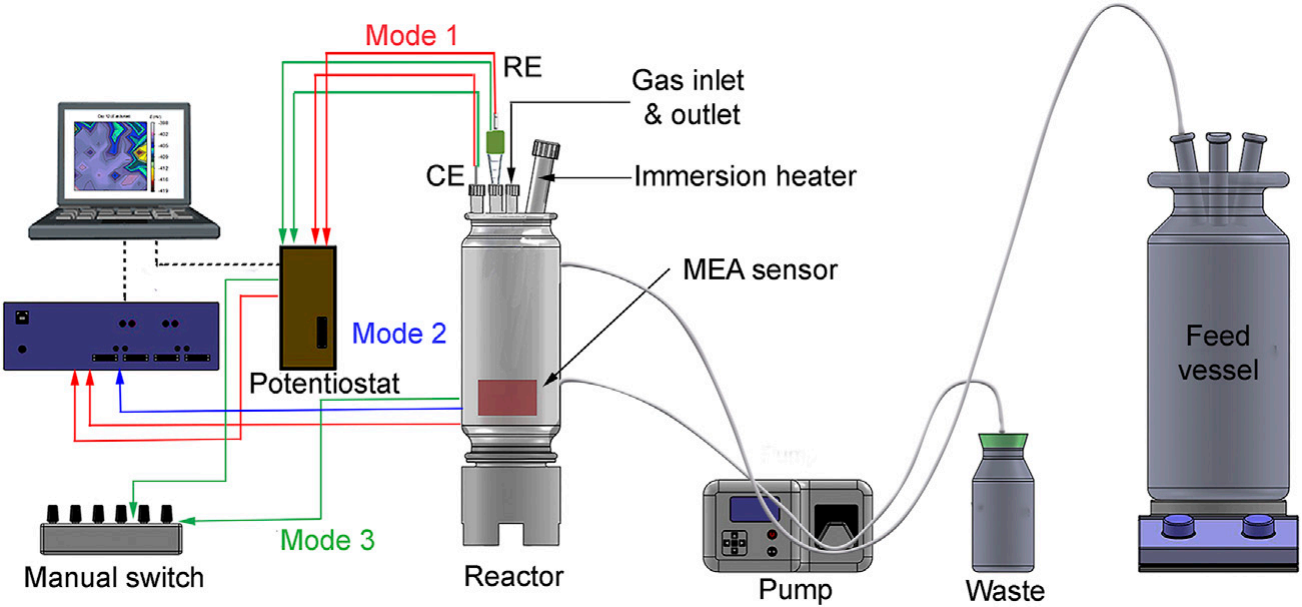
Schematic diagram of the experimental setup for under-deposit microbial corrosion (UDMC) testing using an MEA sensor adapted to operate in three modes. The MEA system is connected to a feed vessel in a continuous flow mode. The counter electrode (CE) and the reference electrode (RE).

An MEA system (CPE systems Pty Ltd) was used to measure galvanic currents and potentials locally. Also, local corrosion rates were calculated from linear polarization (LP) measurements at each wire. The steel sensor which hereafter will be referred to as “the sensor.” The sensor consisted of 100 API X65 pipeline steel electrodes (0.0595 cm^2^ each) electrically insulated and tightly packed with a total surface area of 5.95 cm^2^. Figure 1 shows a schematic diagram of the experimental setup for UDMC evaluation using the MEA system adapted to operate in three different modes (described in Section 2.6). The MEA instrument (blue box in Figure 1) is a pre-programmed auto-switch device and, an ACM AutoZRA in which the sensor is connected. The 100 steel electrodes of the sensor are multiplexed to a 16-bit analog-to-digital converter (ADC) to allow individual measurements. Additional MEA specifications were described elsewhere (Tan and Revie, 2012). The reactor was a custom-made glass cell designed to operate under continuous flow mode. A Teflon base was also designed to mount the sensor in an upright position inside the reactor. The Teflon base, the reactor, and the glass lid were hermetically sealed to ensure anaerobic conditions through the immersion period. The temperature was controlled using a custom-made immersion heater with an external glass sheath. A single junction Ag/AgCl (3 M KCl) reference electrode was placed into a ceramic tip capillary filled with sterile 3 M KCl. The counter electrode was a platinum-coated mesh.

### 2.4 Experimental Setup

The sensor surface was wet ground to 600 grit finish (SiC paper), washed with absolute ethanol, dried with N_2_, and subjected to ultraviolet germicidal irradiation (UVGI) for 15 min. All the non-autoclavable parts of the reactor were immersed in 70% ethanol for 30 min, dried with N_2_ gas and, sterilized by UVGI exposure for a minimum of 15 min. The reactor was assembled and de-oxygenated using a CO_2_/N_2_ gas mixture for 15 min before transferring the CO_2_/N_2_-saturated ASW into the reactor. The MEA terminals of the steel surface were connected to the MEA instrument.

The test conditions were as follows: 1) abiotic test (ASW only), this test was maintained in batch condition (no replenishment of nutrients) through the experimental period of 12 days; 2) biotic test (ASW + bacteria) and 3) corrosion inhibition or CI test (ASW + bacteria + MPY). The sensor was exposed to CI for 18 h (pre-inhibition step) before inoculation of bacteria to allow the uniform formation of the CI film on the surface.

For biotic and CI tests, the reactor was set in batch mode (no replenishment of nutrients) during the first 6 days of exposure to facilitate bacterial settlement. After this period, the reactor was switched to continuous flow mode by connecting it to a feed cell via a peristaltic pump (Figure 1), which allowed continuous replenishment of ASW in the reactor (ASW and inhibited-ASW for the biotic and CI test, respectively). This ASW replenishment was performed to sustain microbial activity throughout the exposure. The dilution rate was 0.005/h-1 equivalent to 250 ml of ASW exchanged every day. Electrochemical monitoring across the MEA was conducted continuously during 12 days of experiments. All the tests were conducted at 40 ± 5°C, maintaining a 20% CO_2_/N_2_ blanket for a total immersion period of 12 days.

### 2.5 Bacterial Enumeration and cATP Measurements

Every two (2) days an aliquot of ASW was collected to estimate planktonic cells of *E. roggenkampii* using a Neubauer chamber and phase-contrast microscopy (Nikon Eclipse Ci-L, Nikon Inc.). An aliquot was also taken to measure cellular adenosine triphosphate (cATP) using the Quench-Gone™ organic modified test kit and a PhotonMaster™ Luminometer (Luminultra Technologies Ltd). This form of ATP serves as a direct indication of total living biomass in suspension. The ASW pH was monitored every 2 days using the Thermo ScientificTM Orion™Star A221 pH portable meter. These analyses were performed in duplicate.

### 2.6 Electrochemical Analysis

Figure 1 shows the schematic diagram of the setup for UDMC testing. The system was operated in three (3) different modes as follows:

#### 2.6.1 Operation Mode 1 (Corrosion Rates and Corrosion Potentials at the Entire Steel Sensor)

The 100 wires or electrodes terminals in the sensor were connected to the MEA instrument (auto-switch device), and this to the Gamry +600 potentiostat as shown by the red dashed lines (Figure 1). In this mode, electrochemical tests were performed by coupling all the micro-electrodes, thus creating a large one-piece working electrode. Therefore, electrochemical measurements reflected the events from the entire sensor. Linear polarisation (LP) was conducted by applying a potential perturbation of ±10 mV vs OCP and a scan rate of 0.5 mV/s. The stabilization period before LP measurements was 30 min, during which the OCP was continuously recorded. The corrosion rates from LP measurements were calculated assuming a Stern-Geary constant of 26 mV (ASTM International, 2014).

#### 2.6.2 Operation Mode 2 (Local Galvanic Currents)

The blue dashed lines in Figure 1 illustrate this operation mode, where the MEA was connected to the auto switch to measure local galvanic currents. In the instrument, the 100 inputs from the were multiplexed to a 16-bit analog-to-digital converter (ADC). A ZRA was used to perform current measurements; thus, no voltage was developed across the inputs. Each electrode was measured for 1,000 milliseconds at a 10 Hz sample rate with a current range of 1 μA–10 mA. Galvanic currents were obtained by performing sequential measurements between each electrode and the remaining 99 electrodes short-circuited. A total of 100 current measurements were recordedand plotted using OriginPro® 2019 to create the current distribution maps. Although these distribution maps contain the 100 measurements recorded individually, once plotted, the map resembles the entire surface of a steel sample with different anodic and cathodic sites across the surface.

#### 2.6.3 Operation Mode 3 (Local Corrosion Potentials and Local Corrosion Rates)

The green dashed lines in Figure 1 describe this operation mode. The MEA terminals were connected to a custom-made manual switcher which in turn was connected to the potentiostat. The manual switcher contains 100 pins that allow individual selection of the electrode in the sensor to be measured by the potentiostat. Similar to mode 1, this mode operated as a three-electrode configuration but this time, recording electrochemical measurements at each electrode in the array (one at a time). LP measurements were performed on each electrode by applying a potential perturbation of ±10 mV vs. OCP and a fast scan rate of 5 mV/s. Similar to operation mode 1, corrosion rates from LP measurements were calculated, assuming a Stern-Geary constant of 26 mV. The OCP at each electrode was recorded for 10 s before performing the LP measurements.

It is acknowledged that the fast scan rate can result in some interference due to measuring capacitance at this faster scan rate. However, this technique was adopted to determine the trend in corrosion rates, and it will be shown later that the measurements correlated well with the potential and galvanic currents measured. Data fitting of each electrode was made using Gamry Echem Analyst, version 7.05, Gamry Instruments, and Inc. A total of 100 corrosion potentials and, 100 corrosion rates values were plotted to create each map using OriginPro®2019.

### 2.7 Visual Inspection and Surface Profilometry

After the immersion period, the reactor was disassembled, and the sensor was washed with ASW and gauze to gently remove the deposit layers formed over the entire steel surface in both biotic and CI tests. Subsequently, the metal was cleaned with absolute ethanol and dried with N_2_ gas. After this step, small deposits remained attached to the sensor. These deposits were removed from the surface using Clarke’s solution following the standard cleaning procedure (ASTM International, 2017). Surface profilometry analysis was conducted using a LaserScan profilometer, Solarius SolarScan non-contact measuring system 200NP (Solarius Inc, United States). The 3D inspection system is equipped with SolarScan NT software version 7.4. The analysis of profiles was performed by step height measurements using the automatic method as described in ISO 5436 standard (ISO 5436, ISO, 2000). The voids above 10 µm deep were considered pits. The analysis included the maximum and average pitting depth and, pit density (number of pits/cm^2^). Pit density was obtained by counting all pits on the entire sensor using the 3D image.

### 2.8 Statistical Analysis

PAST (v3) (Hammer et al., 2001) software was used to analyze statistical differences between biotic and CI tests based on galvanic currents and corrosion potentials recorded at the sensor. The analyzed data comprised ten measurements of each parameter (currents and potentials) recorded at each microelectrode every 2 days (local electrochemistry only). Statistical analysis was also applied to surface pitting data from all tests. Results with *p* values ≤0.05 were established as significantly different. The normality of the data for each variable was assessed by the Shapiro-Wilk test (Shapiro and Wilk, 1965). Then, an analysis of variance (ANOVA) and Tukey’s pairwise comparison (Tukey, 1949; Copenhaver and Holland, 1988) was used to test significant differences in variables with a normal distribution. Permutational multivariate analysis of variance (PERMANOVA) (Anderson, 2001) evaluated significant differences in variables with non-normal distribution.

## 3 Results

### 3.1 Bacterial ATP and, pH Monitoring

**FIGURE 2:**
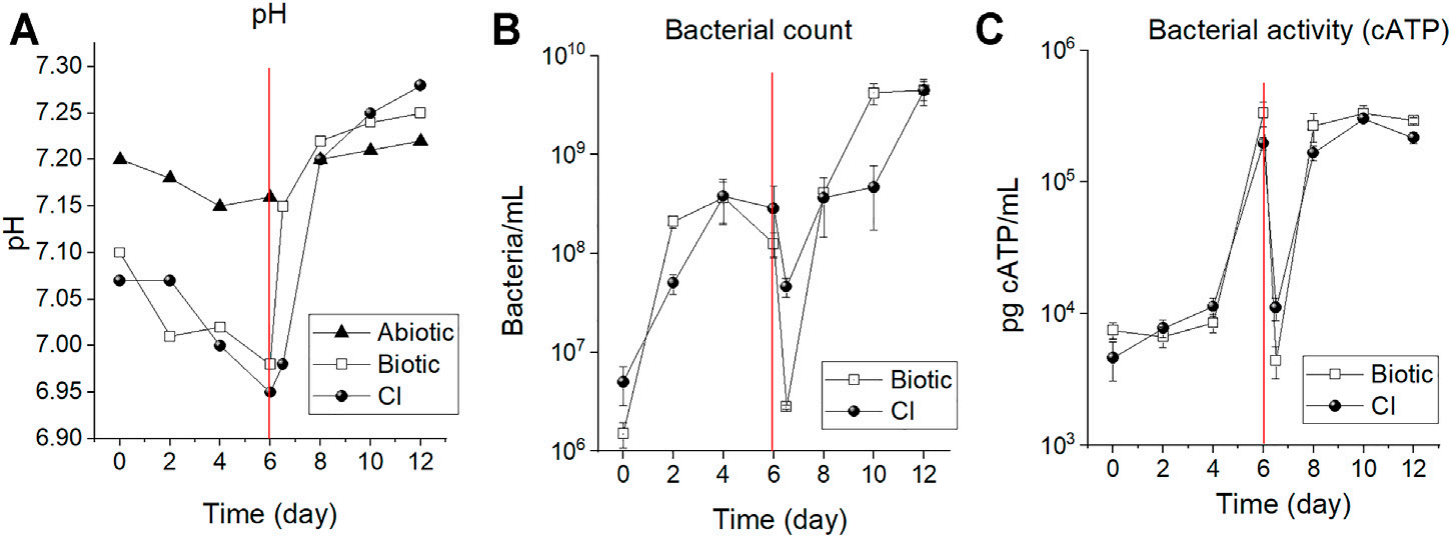
Reactor monitoring system: **(A)** pH; **(B)** bacterial enumeration and, **(C)** cATP measurements of the CO_2_/N_2_ saturated ASW during 12 days of immersion. The dashed line indicates the time in which the reactor was switched to the continuous flow mode by connecting it to the feed cell. The measurements after the dashed line (6.5 days) were performed after 12 h of flow connection with approximately 625 ml of ASW exchanged (i.e., the initial volume of 500 ml + 125 ml).

Figure 2 shows pH, bacterial cell counts, and ATP measurements conducted every two (2) days during the period of immersion. It can be seen in Figure 2A that the pH in the abiotic test remained close to 7.2 for the whole immersion period. However, the pH for the biotic test and CI test gradually decreased during the first 6 days of immersion suggesting accumulation of metabolic by-products in the solution when the reactor was set in a stagnant mode (no flow of fresh solution). -After connection with the feed cell, the pH increased by almost 0.2 pH units due to the replenishment of fresh and neutral solution and removal of acidic microbial metabolites. -. In this continuous-flow mode, the pH remained constant until the end of the immersion. The bacterial enumeration in Figure 2B indicates a gradual increase in cell numbers in test solution from day zero (0) to the fourth day for both tests. The cell numbers in the biotic test increased from 1.5E+06 to 3.6E+08, and the CI test from 5.0E+06 to 2.8E+08 bacteria/mL. From the fourth to sixth day, the culture exhibited a stationary phase until the sixth day, where cell numbers dropped upon start-up of continuous mode due to the removal of planktonic cells from the test solution (dilution rate of 0.25 h^−1^). This drop at 6.5 days of immersion (Figure 2B) reached values of 2.8E+06 and 4.6E+07 bacteria/mL for the biotic and CI test, respectively. After 2 days of continuous replenishment (eighth day), cell density increased because of the addition of growth-limiting nutrients (carbon and nitrogen source) (Madigan et al., 2014).

Similarly, a reduction of the cATP content was observed after 12 h of the reactor being switched to the continuous mode (6.5 days of immersion). The values decreased from 3.3E+05 to 4.4E+03 and from 7.0E+04 to 1.2E+03 for the biotic and CI test, respectively (Figure 2C). Afterward, cATP content increased again for both tests indicating favorable bacterial growth conditions as a result of nutrient replenishment and, elimination of the excess of metabolic waste.

### 3.2 Corrosion Potentials and Corrosion Rates at the Entire Steel Sensor

**FIGURE 3:**
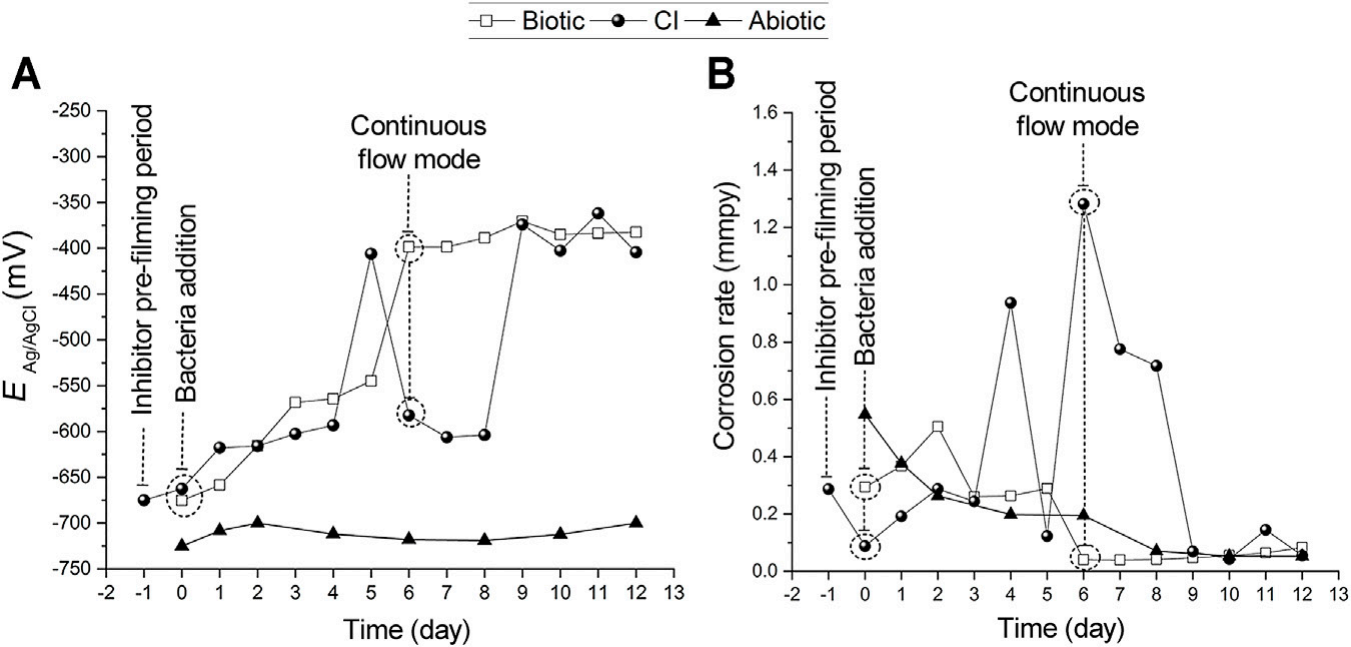
**(A)** Open circuit potential and**, (B)** corrosion rates from polarization resistance measurements recorded at the coupled MEA (operation mode 1) immersed in CO_2_/N_2_ saturated-ASW at 40°C for 12 days. For the CI test, the MPY pre-filming period (∼18 h) is represented as the day (−1) in the *X*-axis. From day zero (0) to the 12th day comprises the immersion period in the presence of bacteria. The dashed line on the sixth day indicates the time in which the reactor was switched to the continuous flow mode by connecting it to the feed cell.

Figure 3 displays corrosion potentials and corrosion rates measurements from LP measurements recorded at the coupled MEA immersed in CO_2_/N_2_ saturated-ASW (operation mode 1 described in Section 2.6). While the potentials in abiotic tests remained steady (−712 ± 9 mV), the potentials in the biotic test and CI test constantly fluctuated during the immersion period (Figure 3A). Looking at Figure 2A results where is a drop in pH over the first 6 days for the Biotic and CI test (before continuous flow started), it can be suggested that this corresponds to the increase in corrosion potential during the same period as shown in Figure 3A. This is expected because the increase in H^+^ should increase the equilibrium potential for the hydrogen evolution reaction or hydrogen ion reduction, 2H^+^ + 2e ↔ H_2_^↑^. In the pre-filming period (18 h of inhibitor contact and before bacteria inoculation) a positive shift of the corrosion potentials from −675 mV to −663 mV (Figure 3A) was observed. This positive shift in potentials after MPY addition has also been reported in previous inhibitor studies (Suarez et al., 2019c).

After bacteria inoculation, the corrosion potentials gradually shifted towards positive values from day zero (0) to the fifth day for biotic test and from day zero (0) to the fourth day for the corrosion inhibition test by +130 mV and +69 mV to values of −545 mV and −593 mV, respectively (Figure 3A). Afterward, the corrosion potentials dramatically shifted again more positively by +146 mV on the sixth day for the biotic test and by +187 mV on the fifth day for the CI test. It can also be noticed in Figure 3A that when the reactor for the biotic test was set to continuous flow mode, the corrosion potentials recorded at the steel MEA remained steady, reaching a maximum value of −371 mV on the ninth day. Contrarily, the steel MEA at the CI test exhibited a considerable positive shift of the corrosion potential by + 229 mV to a value of -374 on the ninth day. On the 11th day of immersion, a maximum corrosion potential value of -362 mV was recorded for the CI test.

The average corrosion rates are shown in Figure 3B. Regarding the biotic test, there was a gradual increase in corrosion rate during the first three (3) days of immersion with a maximum value of 0.5 mmpy recorded on the second day. Then, corrosion rates progressively dropped until the last experimental day with a value of 0.1 mmpy. The abiotic control exhibited low corrosion rates throughout the exposure from 0.6 mmpy on day zero (0) to 0.05 mmpy on the 12th day of immersion.

Figure 3B indicated a reasonable level of protection achieved by MPY in the pre-filming period (18 h contact). The average corrosion rates dropped from 0.3 to 0.1 mmpy upon the addition of MPY. This inhibition performance of MPY has been previously demonstrated at fully deposited-steel surfaces in a CO_2_ environment at 30°C (Pandarinathan et al., 2013; Suarez et al., 2019c). However, after bacteria addition corrosion rates fluctuated, reaching maximum values of 0.9 and 1.3 mmpy on the fourth and the sixth day, respectively. This correlates with the decrease of pH recorded in this period (Figure 2A). Upon continuous flow mode, in this CI test, the average corrosion rates gradually decreased, reaching a corrosion rate value of 0.05 mmpy on day 12 of immersion. Corrosion rates for the biotic test slightly increased and fluctuated the first 6 days after bacteria addition. Considering the drop in pH for this biotic test at this period (Figure 2A) and not marked uniform corrosion rates, it can be suggested a certain level of general protection. It is known that a substantial rate of localized corrosion under a deposit can cause cathodic protection of the non-deposited surface and therefore tends to reduce the rate of general corrosion.

### 3.3 Local Electrochemistry-Abiotic test

**FIGURE 4:**
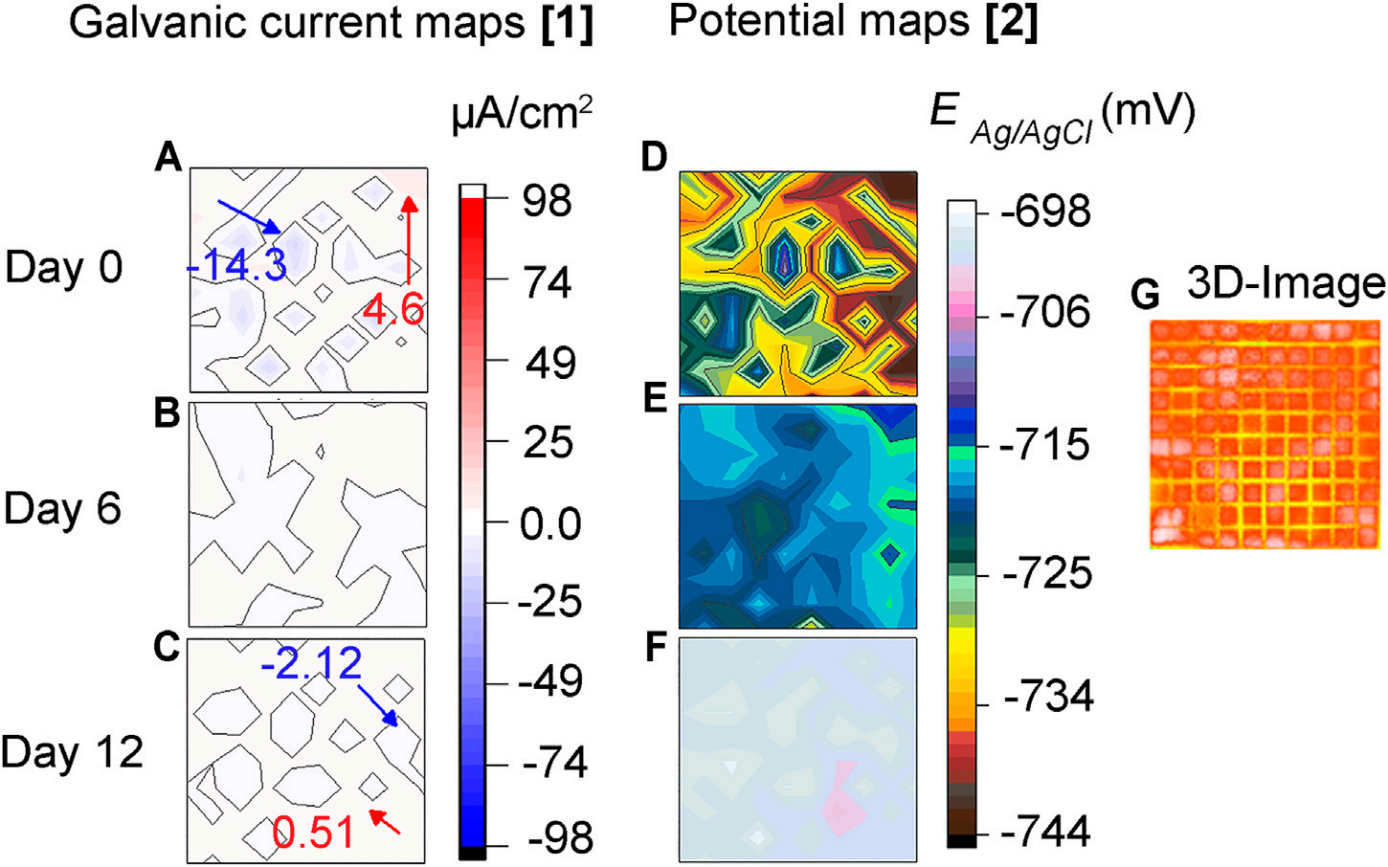
Abiotic test: **(A–C)** Galvanic current, **(D–F)** corrosion potential distribution maps of steel MEA surface immersed in CO_2_/N_2_ saturated-ASW under abiotic or sterile conditions, and **(G)** 3D-image of the entire MEA surface after 12 days of immersion. Inset: maximum anodic and cathodic currents in red and, blue color, respectively.

Figure 4 shows galvanic distribution maps across the sensor under abiotic conditions. It can be seen in Figure 4A that on day zero (0) various cathodic and anodic areas are formed on the surface due to the heterogeneous nature of the corrosion process. These sites continued to change, showing a continuing development in time and space of anodic and cathodic areas (Figures 4B,C). However, the magnitude of these currents is low, and the potential differences across the electrodes are small (Figures 4F,G) indicating low corrosivity of the test solution under abiotic conditions. These results are in agreement with the 3D image in Figure 4D where the sensor looks unaffected and with no apparent signs of localized corrosion.

### 3.4 Local Electrochemistry-Biotic test

**FIGURE 5:**
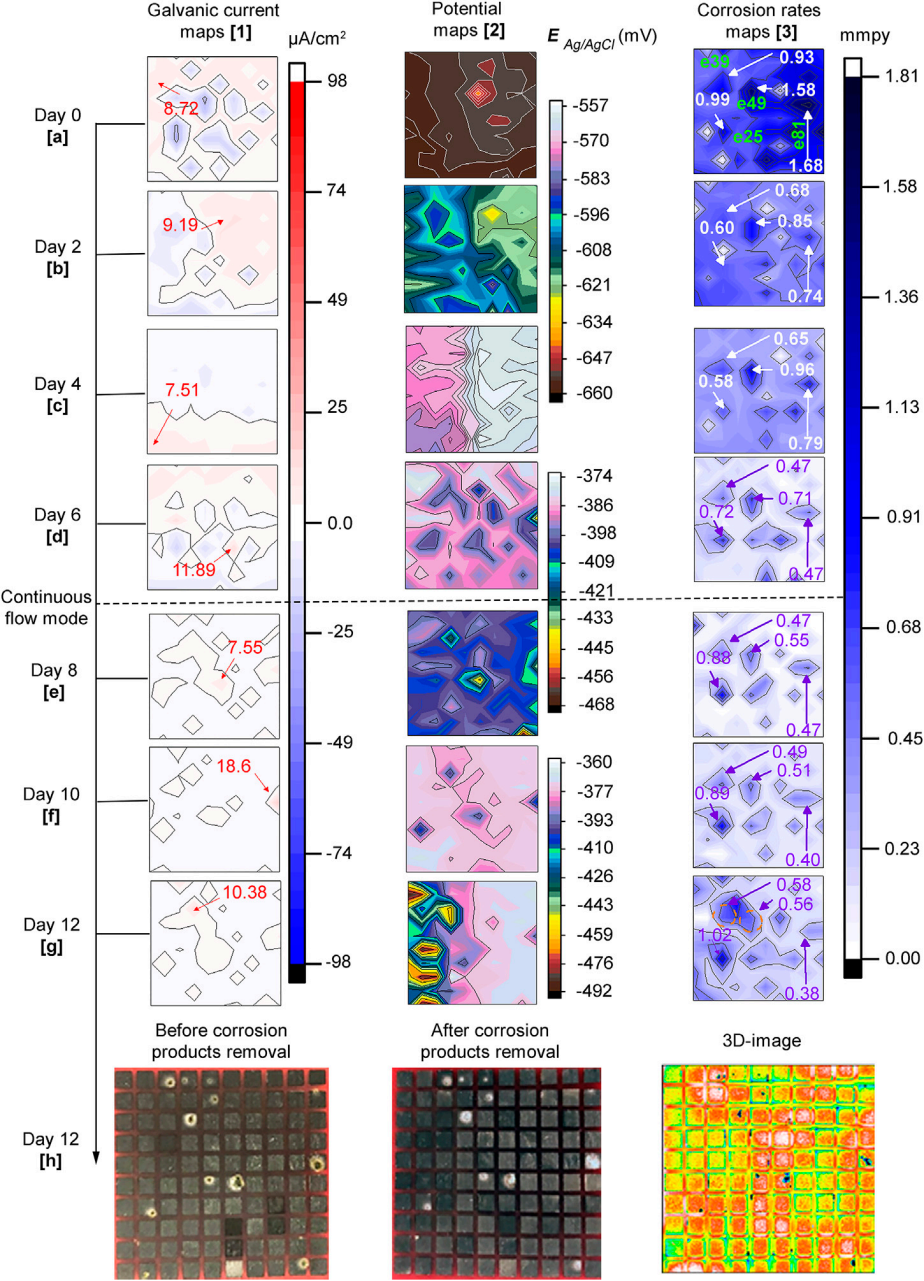
*Biotic test*: **5. 1(A–G)** galvanic currents; **5. 2(A–G)** corrosion potentials and **5. 3(A–G)** corrosion rates distribution maps of the steel MEA immersed in CO2 saturated-ASW and bacteria at 40°C for 12 days. The current maps show the maximum anodic currents in red while for the corrosion rate maps high corrosion rates are shown in violet and white and electrode number in green. Photographs of the entire MEA surface **5.1(H)** before and **5. 2(H)** after corrosion products removal; **5. 3(H)** 3D-image **(front view)** of MEA surface after corrosion products removal.

Figure 5 displays distribution maps of galvanic currents (first column), corrosion potentials (second column) and, corrosion rates (third column) across the sensor in the biotic test. The Figure also shows optical surface images of the sensor before and after the corrosion products were removed and, a 3-D image in the last row. It is important to clarify those areas of the steel surface that recorded net galvanic currents with anodic and cathodic behavior will be referred to as anodic and cathodic areas throughout the text to facilitate the description of the current distribution maps.

The scale shown on the potential maps was set using the maximum, and minimum values recorded at each time to visualize small differences in potential. In general, the distribution of potential is in agreement with galvanic currents and corrosion rates mapped across the surface of the MEA. Sites with higher negative potentials matched the anodic areas and these, in turn, coincided with many of the areas with higher corrosion rates. Also, a general trend of the potentials to shift towards more positive values through time was observed in this biotic test. The maximum and minimum potential values were −660 and −641 mV on day zero (0) and −492 and −360 mV on day 12, respectively. Based on the results shown in Figure 5, the sequence of events that describe UDMC evolution across the steel MEA is presented as follows:

Day zero (0)*:* Net galvanic currents (Figure 5A.1) show different anodic and cathodic areas indicating heterogeneous reactions at the early stages of biofilm adhesion. However, as indicated in Figure 5A.2 small differences of only 19 mV in corrosion potentials across the steel surface were observed. Interestingly, a wide range of corrosion rates from 0.01 to 1.68 mmpy was recorded through the sensor. These local differences in corrosion rates indicated a heterogeneous corrosion process at this initial MIC stage.

Second day: anodic and cathodic areas continued developing in time and space, the region at the top right of the sensor exhibited higher anodic currents than the adjacent area (Figure 5B.1). This coincided with the corrosion potential maps (Figure 5B.2) which had more negative potentials in this region. In addition, the differences in potentials were higher at up to 51 mV at this time compared to the ones recorded on day zero (0). It can also be noticed in Figure 5B.3 that local corrosion rates across the surface of the MEA decreased in magnitude. This observation is particularly noticeable in the regions labeled in the Figure.

Fourth day: Figure 5C.1 shows there was a shift in current distribution across the surface of the electrode array with anodic currents developing in areas that had previously exhibited cathodic currents. Only a maximum potential difference of 24 mV was recorded at this immersion time (Figure 5C.2). Some electrodes in Figure 5C.3 showed higher and lower corrosion rates in similar areas compared to the previous map. These results could be an indication of non-linear localized corrosion evolution under these conditions.

Sixth day: a reversal in galvanic current was again observed, but this time, the anodic currents increased, reaching a maximum value of 11.89 μA/cm^2^. It can be seen in Figures 2B,C that on this day, high values of planktonic cell counts (1.2E+08 bacteria/mL) and ATP content in the solution (3.3E+05 pg/ml) were observed. Thus, it is possible to relate this increase of anodic currents to bacteria presence, probably translated into an efficient metabolic activity leading to local corrosion. The corrosion rate distribution map followed the same trend as the previous map displaying corrosion rates fluctuations at specific locations. However, most of the areas on the sensor showed lower corrosion rates than the ones recorded in previous days.

Eighth day: it can be observed in Figure 5E.1 that there was a decrease in anodic currents across the sensor. At this point, approximately 2 days have passed since the MEA reactor was set to a continuous flow mode (≈1 L of ASW exchanged). Perhaps the reduction of excess metabolic by-products allowed better visualization of localized events on the metal surface. For instance, electrode #35 recorded the highest anodic current, and this coincided with the more negative potential (Figure 5E.2). This potential was 79 mV more negative than the adjacent electrode with a value of −389 mV, showing a good correlation with corrosion rates measured at this electrode with a value of 0.51 mmpy (Figure 5E.3).

Tenth (10th) day: Figures 5F.1,F.2 followed the same pattern as the eighth day. However, the site with a maximum anodic current and a more negative potential is in a different location (electrode # 75). Again the areas locally affected seemed to evolve differently.

Twelfth (12th) day: at this point, 6 days have passed since the reactor was connected in continuous flow mode. The anodic currents were in general, reduced reaching a maximum value of ∼10 μA/cm^2^ at electrode #39 (Figure 5G.1). The potential map (Figure 5G.2), on the other hand, shows a high corrosion potential difference of over 130 mV between the most positive and most negative potential, thus localized corrosion was expected. Corrosion rates of the electrodes labeled in Figure 5G.3 increased, indicating localized corrosion propagation events. Through the immersion period, it was noticed that in some of the labeled electrodes the pits seemed to be formed at different times and evolved at different rates suggesting a non-uniform corrosion evolution. The electrodes started with high corrosion rate values on the first day, and then the values gradually decreased until day 6 of immersion. However, after introducing a continuous flow of test solution in the system, corrosion rates of some of the electrodes increased again, indicating a variable local corrosion process under these conditions.

Another notable finding at the end of the experimental period was the confluence of corroded areas, as indicated in the map for electrodes #39 and #49 indicating an expansion of the local damage. The sensor photographed after biofilm removal and before corrosion product removal (Figure 5H.1), revealed corrosion product deposition randomly across the sensor. The deposits had different sizes and were located in different areas within each electrode or wire. The removal of corrosion products (Figure 5H.2) exposed the depth of the corroded areas or pits. These pits were located precisely underneath the deposits. These observations coincided with the 3D image which revealed locally affected areas in the form of pits (blue-black voids) in the places previously covered by deposits. These results demonstrate that under deposit corrosion occurred as a result of *E. roggenkampii* activity.

### 3.5 Local Electrochemistry-CI Test (Before Bacteria Addition)

**FIGURE 6:**
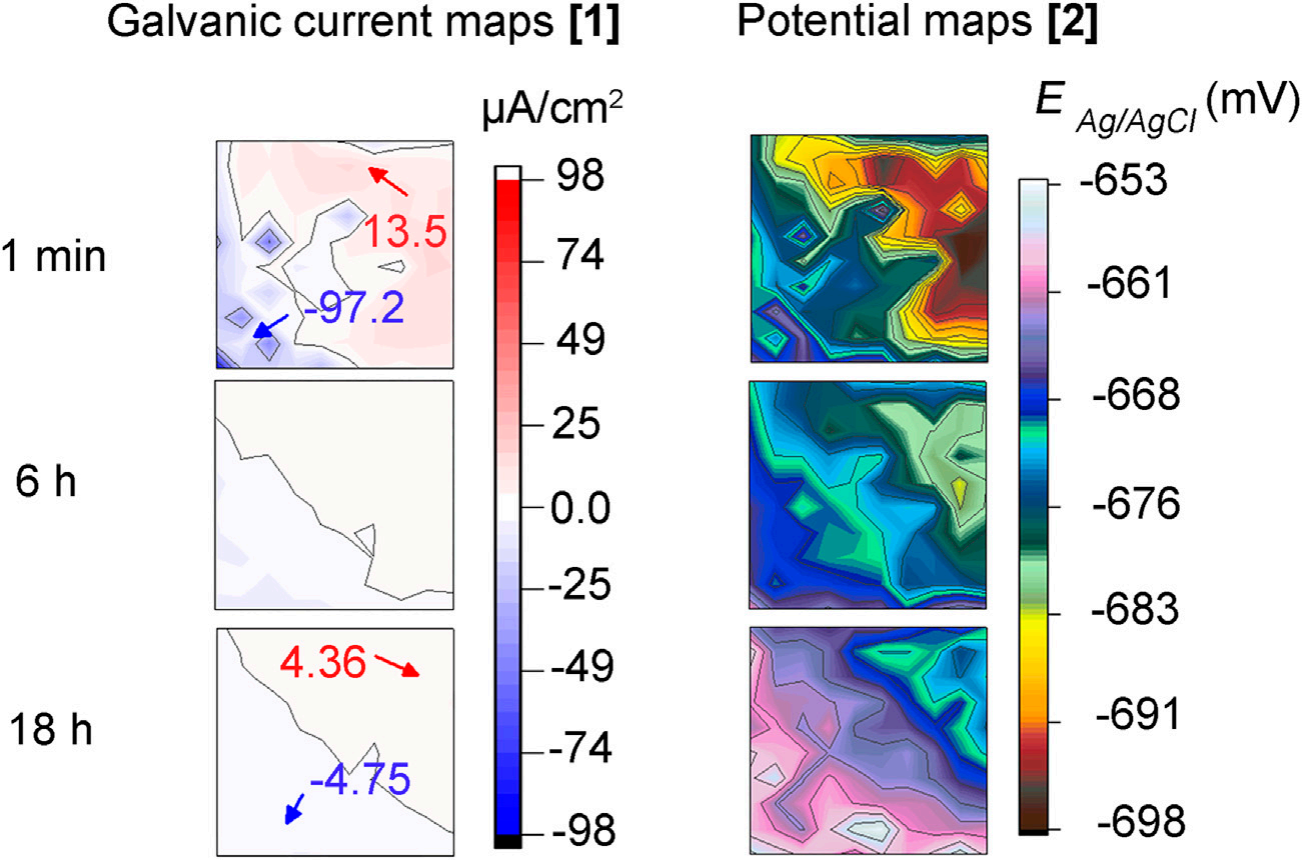
**(A–C)** galvanic currents and, **(D–F)** corrosion potential distribution maps of steel MEA sensor immersed during 18 h in CO_2_/N_2_ inhibited-ASW (0.9 mM of 2-mercaptopyrimidine) before bacteria addition (inhibitor pre-filming period).

Figure 6 shows galvanic current and corrosion potential distribution maps across the MEA at times 1 min, 6 h, and 18 h during the inhibitor pre-filming step before *E. roggenkampii* addition. It can be seen that after 1 min of MPY contact, two (2) anodic and cathodic sites in the top right and bottom left of the sensor, appeared respectively (Figure 6A). These currents are higher than the ones recorded at the MEA immersed in the abiotic test (Figure 4A). This is probably due to the different nature of inhibitor adsorption during early film formation resulting in different anodic and cathodic areas. However, after 6 h (Figure 6B) and 18 h of immersion (Figure 6C) the magnitude of these currents was considerably reduced, indicating acceptable inhibition performance of the MPY. The potential distribution maps (Figures 6D–F) are in agreement with their respective galvanic current maps. The sites of higher negative potentials were situated in the same areas where anodic currents were recorded. After 18 h of immersion (Figure 6F), a more uniform distribution of potential was noticed, probably related to gradual and more uniform filming of the MPY inhibitor across the sensor.

### 3.6 Local Electrochemistry- CI Test (After Bacteria Addition)

**FIGURE 7:**
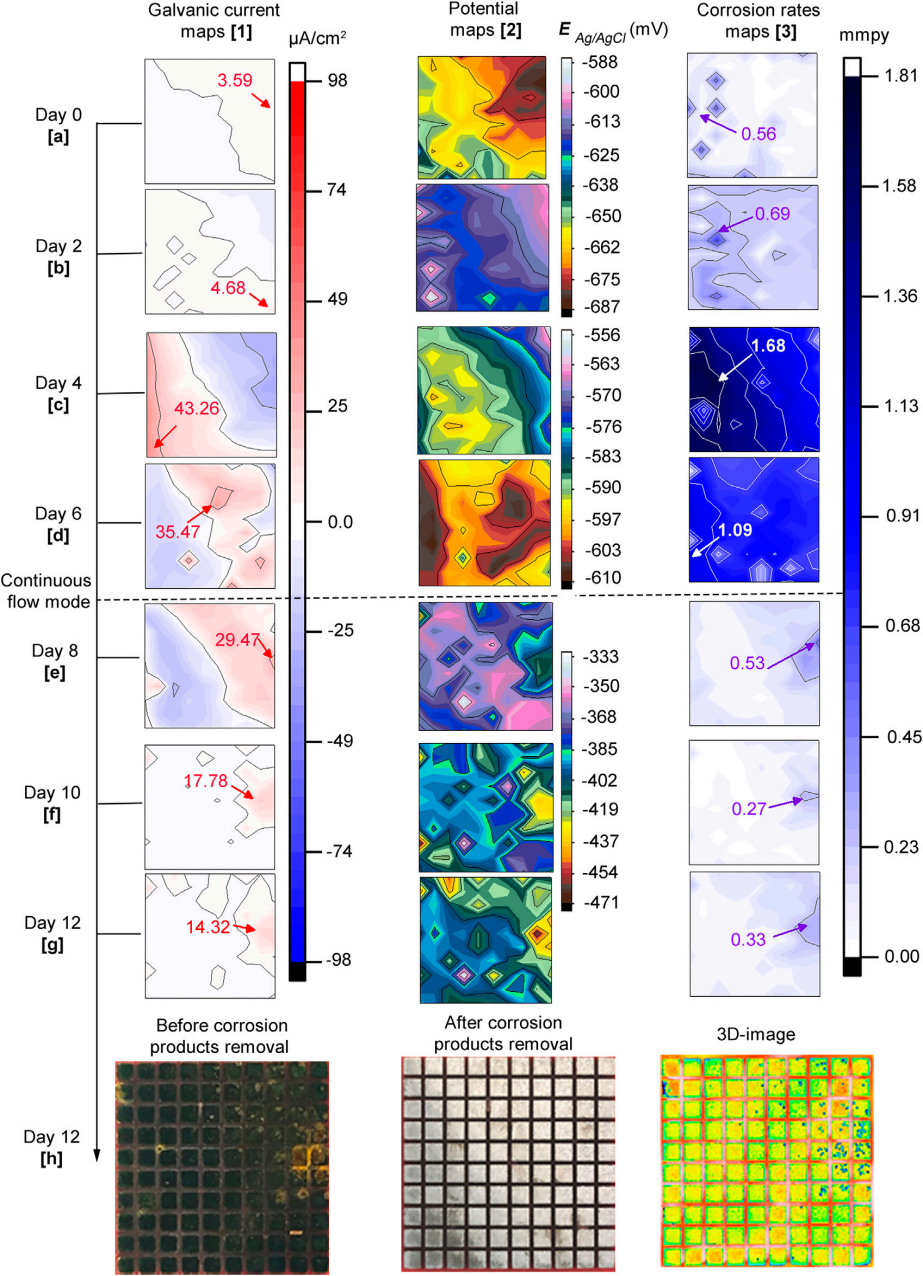
CI test: **7. 1(A–G)** galvanic currents; **7. 2(A–G)** corrosion potentials and; **7. 3(A–G)** corrosion rates distribution maps of the steel MEA sensor immersed in CO_2_ saturated-ASW, bacteria, and corrosion inhibitor (0.9 mM 2-mercaptopyrimidine) at 40°C for 12 days. The current maps show the maximum anodic currents in red while for the corrosion rate maps high corrosion rates are shown in violet and white. Photographs of the entire MEA surface **7.1(H)** before and **7. 2(H)** after corrosion products removal; **7. 3(H)** 3D image of MEA surface.

Figure 7 displays distribution maps of galvanic currents (first column), corrosion potentials (second column), and corrosion rates (third column) across the sensor in the CI test after bacteria addition, at days 0, 2, 4, 6, 8, 10 and 12. The Figure also shows photos of the sensor before and after corrosion products removal and, a 3D-image in the last row. The height scale for the potential maps was set using the maximum and minimum values to visualize small differences in potentials at each map. Similar to the biotic test (Figure 5), the potential maps coincided with most of the galvanic currents and corrosion rate maps. Again, a trend was observed for potentials to move towards positive values in time. The potentials started with values of −687 to −625 mV on day zero (Figure 7A.2) and finalized between −464 and −333 mV on day 12 (over 130 mV difference) (Figure 7G.2). Based on the results in Figure 7, the sequence of events that describe corrosion inhibitor performance in the presence of bacteria is presented as follows:

Day zero (0): no significant changes were detected after bacteria addition. Galvanic current and potential maps remained similar to the ones at the end of the inhibitor pre-filming period (Figures 6C,F). It can be seen in Figure 7A.3 that corrosion rates were low on almost the entire sensor, except for a few areas, e.g. electrode # 16 recorded 0.56 mmpy.

Second day: there was a reversal of galvanic currents and potentials (Figures 7B.1,B.2). The reversal was accompanied by a general increase in corrosion rates corresponding to those regions of anodic currents at more negative potentials.

Fourth day: at this time, the magnitude of galvanic currents, potentials, and corrosion rates increased considerably in areas previously established on the second day reaching 1.68 mmpy at electrode # 26 (Figures 7C.1–3).

Sixth day: galvanic currents and potentials reversed again, but the anodic currents diminished in magnitude (Figures 7D.1,D.2). Corrosion rates decreased to some extent reaching a maximum value of 1.09 mmpy at electrode #2.

Eighth day: The replenishment facilitated the visualization of localized corrosion of the steel across all the distribution maps. This was probably due to the exchange of corrosive metabolites by fresh inhibited ASW, which made it possible to differentiate local corroded areas from adjacent non-corroded areas. Figure 7E.1 shows that after 2 days of continuous flow, the anodic currents expanded covering the top right corner of the sensor. It can also be noticed that there was a marked difference in potentials (120 mV) between the more negative and positive values. The more negative potential (−543 mV) was recorded at electrode #99 (Figure 7E.2), which also had the highest anodic current (Figure 7E.1) and corrosion rate (Figure 7E.3), with values of 29.47 μA/cm^2^ and 0.53 mmpy, respectively. A general reduction of corrosion rate values was evident across the MEA probably due to the replacement of corrosive metabolites by fresh solution with more inhibitors, making it possible to have a more unobstructed view of the local corrosion events.

Tenth (10th) day: It is evident in Figures 7F.1–3 that the three (3) maps are in good agreement. At this stage, almost the total amount of the inhibited ASW (∼2 L) was replenished. Again, potential differences on the surface continued to increase, but this time, a 132 mV difference was recorded between the maximum and minimum values. Instead, the maximum corrosion rate value was only 0.27 mmpy at electrode # 81.

Twelfth (12th) day: Similar to the maps recorded on the 10th day of immersion (Figures 7F.1–3), the maps of the different parameters were congruent with each other (Figures 7F.1–3). However, electrode # 81 had a higher corrosion rate, indicating a localized corrosion propagation process. Figure 7H.1 shows a photograph of the sensor before corrosion products removal and after biofilm cleaning. Similar to the biotic test, corrosion products were also found to be deposited on the steel sensor. These deposits exhibited smaller sizes compared to the deposits found in the biotic test. After corrosion products were removed (Figure 7H.2), pits were exposed underneath the deposits (3D-image in Figure 7H.3). The most affected area (top right corner of the maps and photos) at the end of the experimental period coincided with the initial electrochemistry in the CI pre-filming period. Similar to the biotic test, this observation in the CI test suggested that the first pattern of the electrochemistry on the steel surfaces influenced the later localized corrosion process in the presence of bacteria.

**FIGURE 8:**
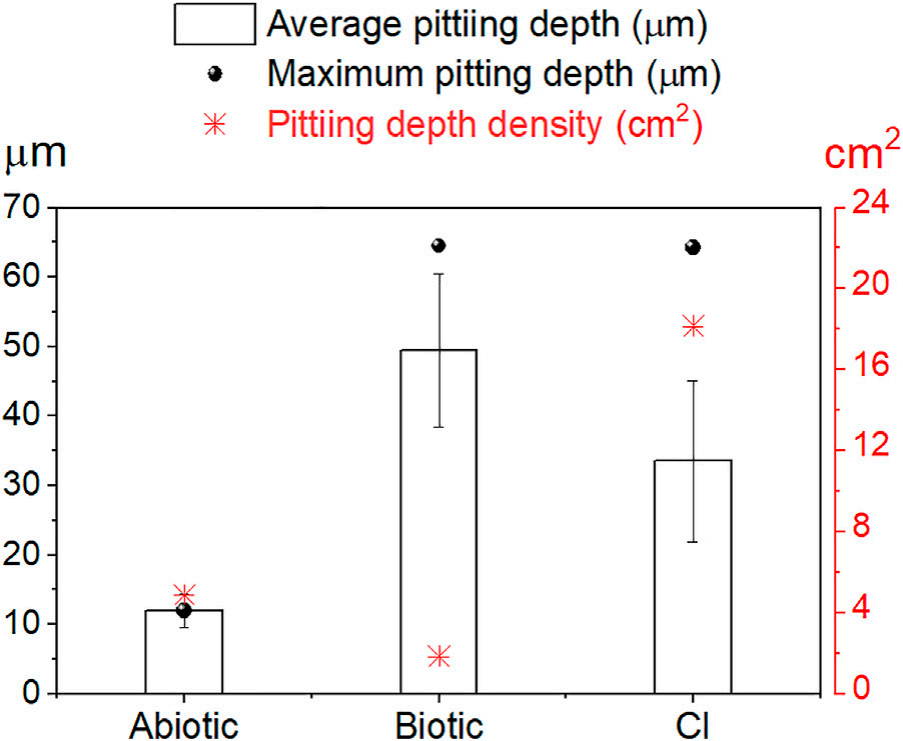
Localized corrosion results by 3D-laser scanning profilometry. Average, maximum pitting depth **(left *y*-axis)** and pit depth density **(right *y*-axis)**. The average pitting depth was obtained using all deepest points measured on each sample. Pit density is the number of pits/cm^2^.

Figure 8 shows the results of the average and maximum pitting depth and pit density from 3D-laser scanning profilometry. Average and maximum pitting depth results indicated a considerable localized attack in the presence of *E. roggenkampii* with a maximum pitting depth of 65 µm in the biotic test and 64 µm in the CI test; the abiotic test exhibited a maximum pitting depth of 12 µm. MPY inhibited average corrosion recording 0.05 mmpy on day 12th (Figure 3B). However, this sulfur-containing compound did not inhibit localized corrosion in the presence of bacteria, which represents a significant concern to industry (Figure 8).

Although a slightly higher average pitting depth resulted on the sensor in the biotic test, the surface in the CI test still suffered considerable localized corrosion damage. In fact, the surface in this CI test showed higher pit density (18.2 pits/cm^2^) compared to the biotic test (1.8 pits/cm^2^). The number of pits can be easily visualized on the 3D-image in Figure 5H.3 for the biotic test and in Figure 7H.3 for the CI test. The abiotic test, on the other hand, did not show distinct pits. The sensor immersed in sterile conditions looked more uniform than both biotic tests, suggesting that no localized corrosion occurred in the absence of bacteria (Figure 4D).

### 3.7 Comparisons of Local Electrochemistry and Localized Corrosion Across the Different Tests

Statistical analysis of the localized corrosion data for the biotic and CI tests confirmed significant differences in the type of localized corrosion (*p* ≤ 0.05, PERMANOVA). Values and statistical details per day are summarized in the Supplementary Table S1. These statistical differences were related to the presence of the corrosion inhibitor (MPY), leading to a different evolution of localized corrosion on the steel-MEA. One way-ANOVA and Tukey’s pairwise tests (Supplementary Table S2) revealed that the differences in average pit depths between abiotic, biotic, and CI tests were significant (*p* ≤ 0.05). Aside from the evident difference between abiotic and biotic systems, these results demonstrated that the extent of localized corrosion was also significantly different when the CI (MPY) was present.

## 4 Discussion

### 4.1 Under-Deposit Microbial Corrosion

This study assessed carbon steel corrosion by *E. roggenkampii*, a bacterium capable of conducting iron oxidation coupled with nitrate reduction in anaerobic conditions. In this work, two types of corrosion were assessed by the MEA system, the galvanic effects reflected in galvanic currents maps caused by potential differences on the surface of the MEA and self-corrosion due to direct attack of the electrolyte on the steel electrodes as shown in the corrosion rate maps. The galvanic current and corrosion rate distribution maps were in agreement with the local visual areas of metal corroded and the corresponding photographs and 3D-images. Experimental results in both biotic and CI tests demonstrated that *E. roggenkampii* influenced localized corrosion by production and accumulation of biogenic deposits on the steel surface. The presence of pits at the steel surface located exactly beneath deposits on the metal surface indicates the occurrence of UDC (Supplementary Figure S1).

Most of the microbial corrosion mechanisms involving iron oxidation have been described for oxygenic habitats, i.e. aerobic corrosion (NACE International, 2018). Overall, is known that in the presence of oxygen the area under the deposit will corrode due to the formation of an oxygen concentration cell. The area under the deposit because it is depleted of oxygen forms the anode while the area outside the deposit, rich in oxygen, behaves as the cathode. The colonies of type of bacteria such as iron-oxidizing bacteria (FeOB) in the presence of oxygen, the area underneath bacterial becomes oxygen-depleted acting as an anode, whereas the area outside of the colonies, where oxygen concentrations are higher than inside, support the cathodic half-reaction (Little et al., 1992). An electrochemical potential difference is eventually developed between the two regions, resulting in the dissolution of the underlying metal. Consequently, dissociated metal ions form ferrous hydroxides, ferric hydroxide, and iron-containing minerals (Gu et al., 2011). Also, some iron-oxidizing bacteria species from the genus *Enterobacter* have been associated with corrosion of carbon steel in aerobic conditions (Ashassi-Sorkhabi et al., 2012).

Contrary, in CO_2_ environments, under deposit type corrosion does not normally occur because the deposit shields and protects the underlying surface from corrosion. In the present study, the presence of bacteria has reversed this trend causing the area directly under the deposit to corrode. *E. roggenkampii,* through its iron-oxidizing/nitrate-reducing metabolism, induced the formation of biogenic deposits on the metal surface, a process that resulted in UDC in a CO_2_ environment. The oxidation of ferrous iron (Fe^2+^) and the subsequent precipitation of ferric iron (Fe^3+^) oxides has been shown to result in localized corrosion underneath deposits (Rao et al., 2000). This process was evidenced by the distribution maps with anodic areas, high corrosion rates, and more negative potentials in areas underneath deposits. In agreement with the electrochemical data, the pits were located exactly beneath deposits. Iron oxides deposited on the steel surfaces can drive a localized corrosion attack by acidification underneath deposits acting as anodes and the area outside deposits as cathodes. For instance, Yong *et al.* (Kim and Kim, 2017) observed pits on carbon steel covered with a rust layer of goethite (α-FeOOH). The author stated that α-FeOOH was transformed into Fe_3_O_4_ leading to corrosion in developed porous defects creating separated reactions, acidification in the anode due to FeCl_2_ presence, and NaOH formation in the cathodic region.

Besides the Fe^3+^ oxides deposition as responsible for the localized corrosion process, it can also be suggested the occurrence of electrical MIC (EMIC) by FeONRB. Previous non-corrosion-related works have demonstrated that some nitrate reducers can use zero-valent iron (Fe^0^) as the only energy source. Those studies based on bioelectrochemical systems (BES) have achieved numerous advances in the understanding of extracellular electron transfer (EET) mechanisms by NRB. For instance, denitrifying biocathodes have been demonstrated to be efficient in removing nitrate from contaminated waters (Ghafari et al., 2008; Kondaveeti et al., 2014; Pous et al., 2014; Molognoni et al., 2017; Jiang et al., 2018). Similarly, the use of nano-zero valent iron (nZVI) as co-electron donors for heterotrophic/autotrophic denitrification has found a better nitrate removal efficiency in groundwaters (Biswas and Bose, 2005; Hu et al., 2018).

In recent years, many studies have shown that a vast list of microorganisms is capable of extracting electrons directly from the steel, which negatively affects metal surfaces leading to corrosion. Sulfate-reducing bacteria (Yu et al., 2013; Enning and Garrelfs, 2014; Kim et al., 2015; Zhang et al., 2015) and nitrate reducers such as *Shewanella* and *Geobacter* species (Mehanna et al., 2009; Xu et al., 2013; Jin et al., 2019; Tang et al., 2021) have been widely studied as causative EMIC microorganisms able to corroded both stainless and carbon steel materials.

### 4.2 Corrosion Inhibitor Performance in the Presence of *E. roggenkampii* (CI Test)

This work also determined the inhibition performance of 2-Mercaptopyrimidine (MPY), before and after the addition of bacterial cells. The distribution maps showed that CI protected the steel surface against localized corrosion before the addition of bacteria cells. However, MPY was unable to prevent corrosion in the presence of bacteria (Supplementary Figure S1).

Drawing a comparison between biotic and CI tests, in terms of average corrosion, the shiny appearance of the steel after corrosion products removal in the CI test compared to the black and opaque steel surface in the biotic test indicates that MPY exerted a level of uniform corrosion protection in the presence of bacteria. However, the surface was locally affected by microbial activity leading to pitting corrosion, reaching similar values of maximum pitting depth (64 µm) compared to the biotic test (65 µm) (Supplementary Figure S2).

Although microbial activity locally affected steel surfaces of both biotic and CI tests, there were some noticeable corrosion differences between them. In the CI test, the overall pit size was smaller than the biotic test. However, pit density was considerably higher when the inhibitor was present. Results also suggest that this compound controlled the extent of localized corrosion as indicated by a lower average pitting depth in the CI test (33.5 µm) than the biotic tests (49.5 µm). This was also supported by statistical analysis that revealed differences in local electrochemistry and localized corrosion between tests (biotic and CI test).

The pyrimidine derivative compound, 2-Mercaptopyrimidine has been reported as high-performance CI in the presence of silica sand, aluminum oxide, and calcium carbonate deposit (Suarez et al., 2019c). This organic, film-forming CI consists of a polar molecule with the S and N atoms forming negative and positive ends respectively of the dipole (Pandarinathan et al., 2013). In this work, MPY showed good performance after 18 h of contact with the steel MEA and before bacteria injection (pre-filming period). However, after 2 days of bacteria addition, *E. roggenkampii* compromised the corrosion protection exerted by this compound. Organic film-forming CIs adsorb onto the metal-solution interface. Thus, it is reasonable to suggest that the adhesion and colonization of *E. roggenkampii* onto the surface destabilized the CI film. This can be correlated with cATP values obtained from the fourth (11,305 pg/ml) to the sixth (195,593 pg/ml) day of exposure, which indicates considerably high microbial activity in reactors during that period.

Some authors claimed (Videla et al., 2000) (Rajasekar et al., 2007) that organic film-forming CIs used in oil and gas systems could be affected by microbial degradation, i.e., microbes can use CIs as carbon sources. Results from this research suggest that the activity of *E. roggenkampii* led to the local formation of iron mineral deposits followed by UDC occurrence even in surfaces pre-filmed with MPY. The acidification of deposited areas induced local electrochemical changes underneath deposits (anodic areas), which could have resulted in a local breakdown of the CI film.

This study demonstrates that the MEA system is a suitable tool to detect, evaluate and monitor localized corrosion phenomena and the efficiency of corrosion inhibitors under MIC and UDC scenarios. Likewise, these results highlight the potential for microorganisms to compromise the efficiency of corrosion inhibitors when conditions supporting microbial activity are present. Therefore, this work underlines the importance of including microbial constituents in corrosion inhibition tests to qualify inhibitor compounds before their application in both industrial and natural environments.

## 5 Conclusion

*Enterobacter roggenkampii,* through its iron-oxidizing capabilities coupled to nitrate reduction, led to deposit formation and under-deposit corrosion of carbon steel in anaerobic conditions. Electrochemical and surface analysis results demonstrated that anodic sites developed beneath deposits, with corresponding high local corrosion rates, more negative potentials, and pitting corrosion in under-deposit areas. Corrosion rates distribution maps indicated that local events initiated and evolved differently with time over the carbon steel surface. 2-Mercaptopyrimidine (MPY), a film-forming corrosion inhibitor did not prevent localized corrosion in the presence of *Enterobacter roggenkampii*. Deposits generated by the metabolic activity of the bacterium created an aggressive acidic environment (anodic areas underneath deposits) that resulted in pitting corrosion under deposits. In the presence of this marine bacterium the MPY inhibitor film, previously adsorbed on the metal surface, was destabilized, and pitting corrosion initiated on the metal surface. The ability of the MEA to locally assess the corrosion process, galvanic effects and, corrosion potentials across the metal surface demonstrated its suitability to detect, evaluate and monitor under-deposit corrosion and MIC as well as to investigate the efficiency of corrosion inhibitors in complex environments involving deposits and microorganisms.

## Data Availability

The original contributions presented in the study are included in the article/Supplementary Material, further inquiries can be directed to the corresponding author.
