# Supplementary material for: In Situ Investigation of Under-Deposit Microbial Corrosion and its Inhibition Using a Multi-Electrode Array System
Source: Front Bioeng Biotechnol. 2022 Jan 10;9:803610. doi: 10.3389/fbioe.2021.803610 (PMC8784807; doi:10.3389/fbioe.2021.803610)
Supplement: Supplementary file 1 [file DataSheet1.zip › Data sheet/Table 1.DOCX]

Supplementary Figure 1


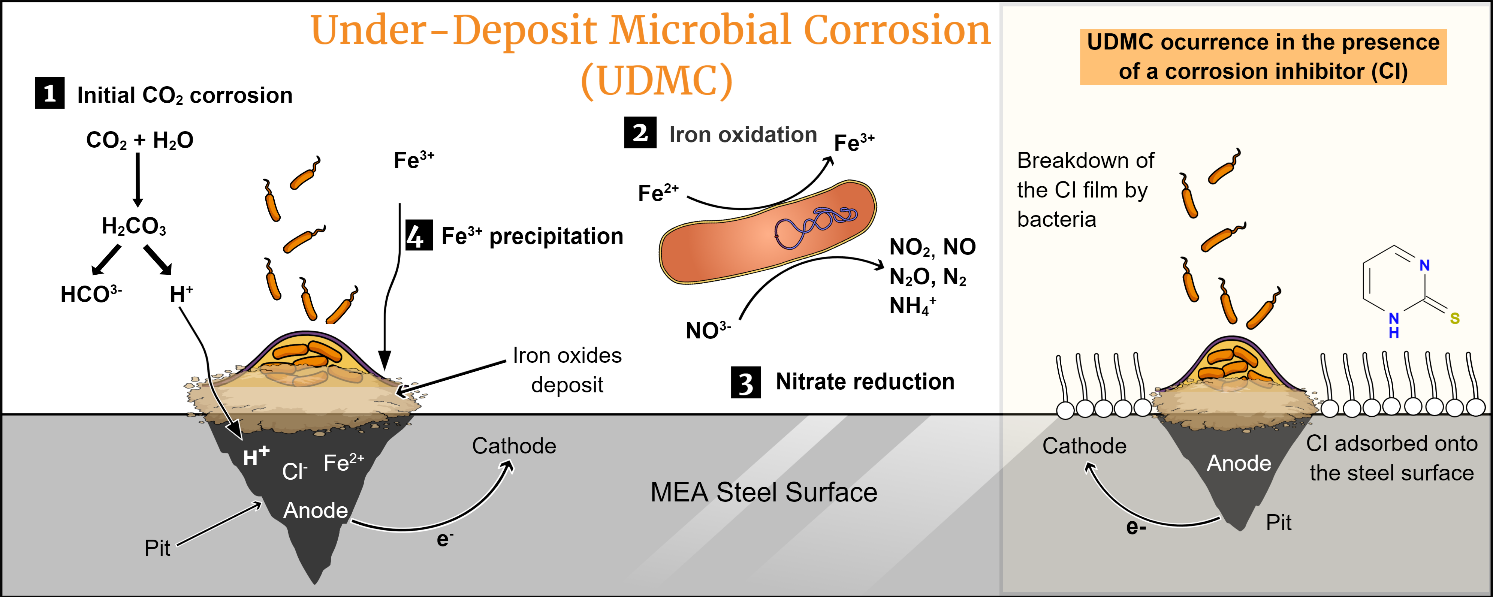


**Supplementary Figure 1.** Schematic representation of under-deposit microbial corrosion (UDMC) on steel surfaces. The pits at the steel surface are located exactly beneath deposits on the metal surface. Left panel: localized corrosion by production and accumulation of biogenic deposits on the steel surface by *E. roggenkampii*. Right panel: UDMC occurrence in the presence of an organic film-forming corrosion inhibitor, 2-Mercaptopyrimidine (MPY).
