# Supplementary material for: In Situ Investigation of Under-Deposit Microbial Corrosion and its Inhibition Using a Multi-Electrode Array System
Source: Front Bioeng Biotechnol. 2022 Jan 10;9:803610. doi: 10.3389/fbioe.2021.803610 (PMC8784807; doi:10.3389/fbioe.2021.803610)
Supplement: Supplementary file 1 [file DataSheet1.zip › Data sheet/Table 2.DOCX]

Supplementary Table 1

# Table 1. Permutational analysis of variance (PERMANOVA) to calculate significant differences between biotic and CI test based on galvanic and corrosion potential measurements recorded at the MEA sensor.

| **Day** | **Permutation N:** | **Total sum of squares:** | **Within-group sum of squares:** | **F:** | **p (same):** |
| --- | --- | --- | --- | --- | --- |
| *Galvanic currents (Biotic vs. CI test)* | | | | | |
|  |  |  |  |  |  |
| 0 | 9999 | 1.26E+08 | 1.02E+06 | 2203 | 0.0001 |
| 2 | 9999 | 1.00E+08 | 6.23E+05 | 2871 | 0.0002 |
| 4 | 9999 | 5.85E+08 | 1.39E+06 | 7570 | 0.0001 |
| 6 | 9999 | 4.14E+08 | 3.78E+07 | 179.2 | 0.0001 |
| 8 | 9999 | 3.75E+08 | 1.68E+07 | 384.8 | 0.0002 |
| 10 | 9999 | 3.78E+07 | 1.10E+07 | 43.57 | 0.0002 |
| 12 | 9999 | 3.74E+07 | 1.07E+07 | 44.93 | 0.0001 |
| *Corrosion potentials (Biotic vs. CI test)* | | | | | |
|  |  |  |  |  |  |
| 0 | 9999 | 1.09E-01 | 7.96E-03 | 228.2 | 0.0001 |
| 2 | 9999 | 1.44E-01 | 3.86E-03 | 653.4 | 0.0001 |
| 4 | 9999 | 2.76E-01 | 8.08E-05 | 61410 | 0.0001 |
| 6 | 9999 | 2.17E+01 | 4.34E-01 | 882.3 | 0.0001 |
| 8 | 9999 | 6.24E-01 | 5.44E-03 | 2046 | 0.0001 |
| 10 | 9999 | 2.87E-01 | 2.52E-03 | 2028 | 0.0001 |
| 12 | 9999 | 1.11E+00 | 3.93E-03 | 5080 | 0.0001 |
|  |  |  |  |  |  |
