# Supplementary material for: In Situ Investigation of Under-Deposit Microbial Corrosion and its Inhibition Using a Multi-Electrode Array System
Source: Front Bioeng Biotechnol. 2022 Jan 10;9:803610. doi: 10.3389/fbioe.2021.803610 (PMC8784807; doi:10.3389/fbioe.2021.803610)
Supplement: Supplementary file 1 [file DataSheet1.zip › Data sheet/Table 3.DOCX]

Supplementary Table 2

# Table 2. Analysis of variance (ANOVA) and Tukey-Kramer tests used to identify significant differences between all tests based on average pit depths.

| **ANOVA (Abiotic vs. Biotic)** | | | | | |
| --- | --- | --- | --- | --- | --- |
| Test for equal means |  |  |  |  |  |
|  |  |  |  |  |  |
|  | *Sum of sqrs* | *df* | *Mean square* | *F* | *p (same)* |
| Between groups: | 12017.7 | 1 | 12017.7 | 385.5 | 5.39E-24 |
| Within groups: | 1434.19 | 46 | 31.178 | Permutation p (n=99999) |  |
| Total: | 13451.9 | 47 | 1.00E-05 |  |  |
|  |  |  |  |  |  |
| *Components of variance (only for random effects):* | | | | | |
| Var(group): | 706.823 | Var(error): | 31.178 | ICC: | 0.957753 |
|  |  |  |  |  |  |
| omega2: | 0.889 |  |  |  |  |
|  |  |  |  |  |  |
| Levene´s test for homogeneity of variance, from means | p (same): | 1.68E-06 |  |  |  |
| Levene´s test, from medians | p (same): | 2.64E-06 |  |  |  |
|  |  |  |  |  |  |
| Welch F test in the case of unequal variances: F=125.8, df=10.29, p=4.277E-07 |  |  |  |  |  |
| **Tukey's pairwise** | | | | | |
|  | *Abiotic* | *Biotic* |  |  |  |
| **Abiotic** |  | **0** |  |  |  |
| Biotic | 27.77 |  |  |  |  |
