# Supplementary material for: In Situ Investigation of Under-Deposit Microbial Corrosion and its Inhibition Using a Multi-Electrode Array System
Source: Front Bioeng Biotechnol. 2022 Jan 10;9:803610. doi: 10.3389/fbioe.2021.803610 (PMC8784807; doi:10.3389/fbioe.2021.803610)
Supplement: Supplementary file 1 [file DataSheet1.zip › Data sheet/Table 4.DOCX]

Supplementary Figure 2


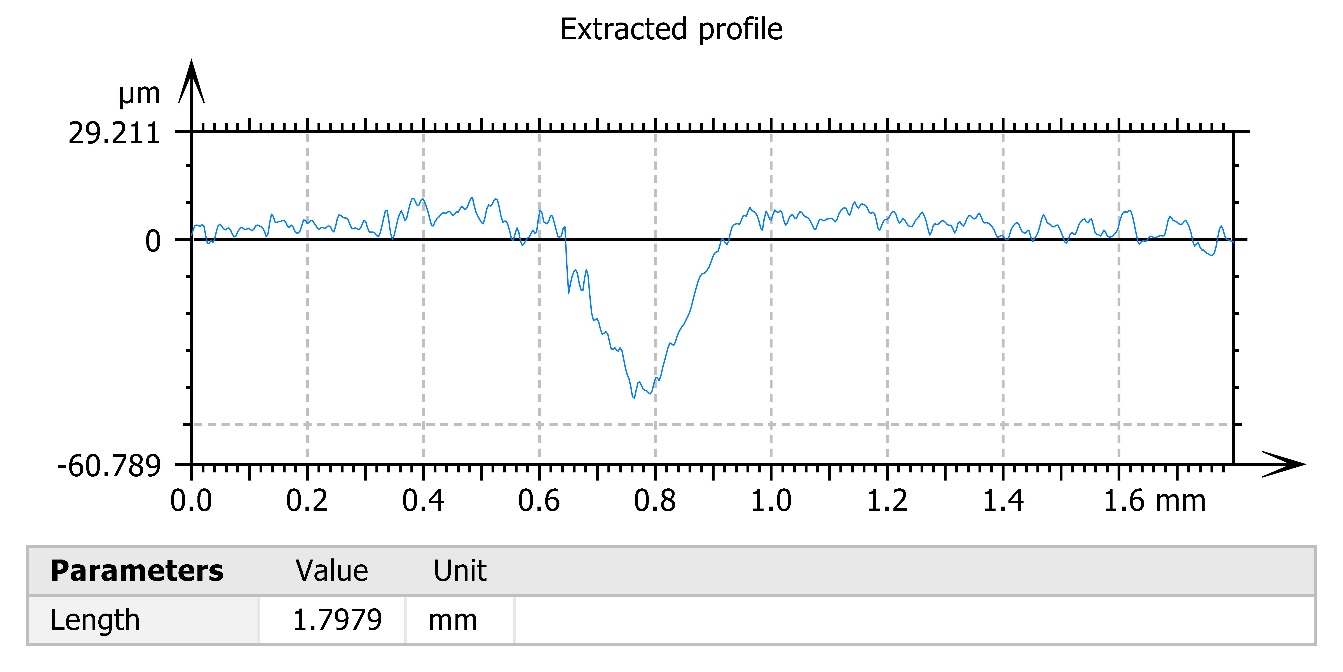


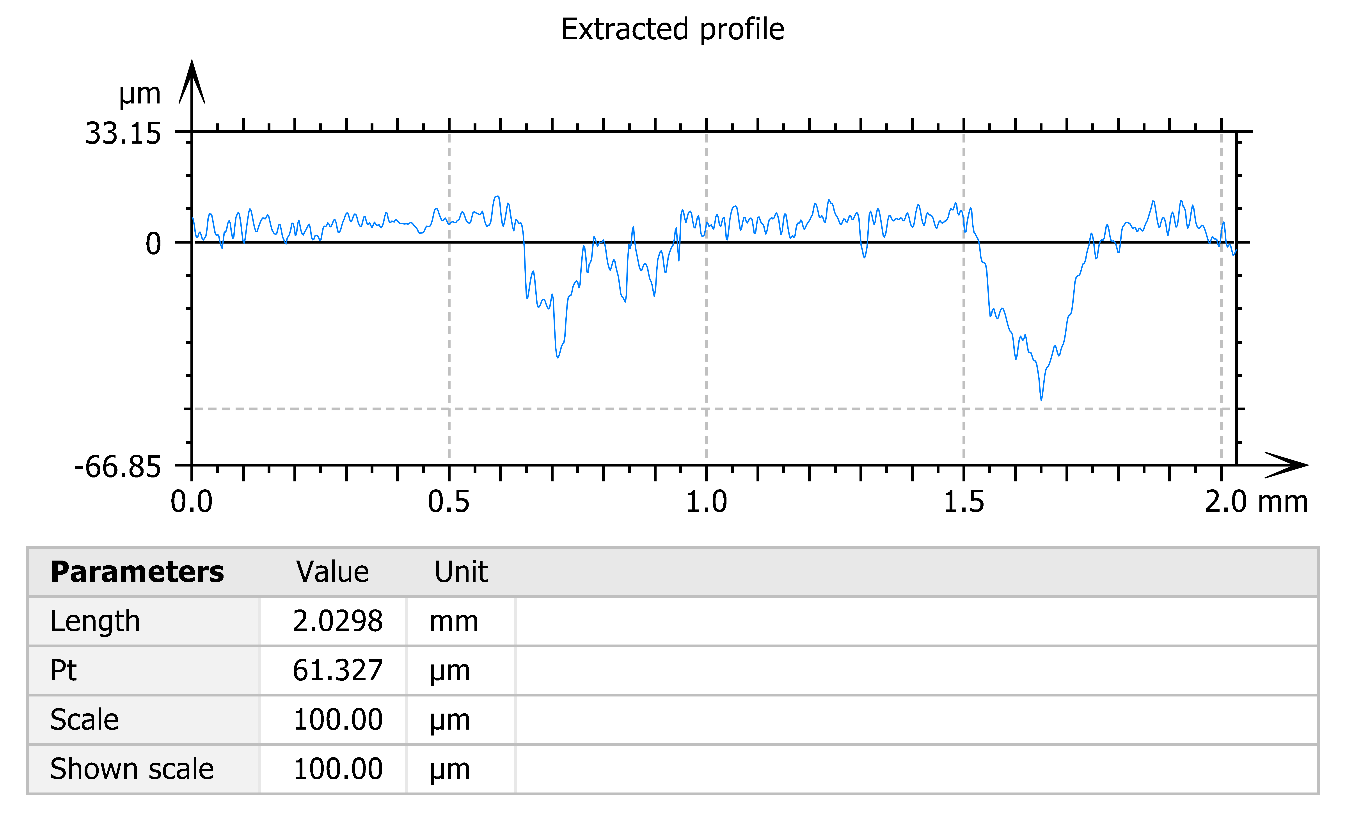


**Supplementary Figure 2.** Extracted profiles of the deepest pits (electrodes #38 and # 52 from biotic and CI tests, respectively).
